# The correlations between Th1 and Th2 cytokines in human alveolar echinococcosis

**DOI:** 10.1186/s12879-020-05135-y

**Published:** 2020-06-15

**Authors:** Xiao Ma, Xuefei Zhang, Jia Liu, Yufang Liu, Cunzhe Zhao, Huixia Cai, Wen Lei, Junying Ma, Haining Fan, Jianye Zhou, Na Liu, Jingxiao Zhang, Yongshun Wang, Wei Wang, Peizhen Zhan, Xiongying Zhang, Qing Zhang, Kemei Shi, Peiyun Liu

**Affiliations:** 1 Qinghai Institute for Endemic Disease Prevention and Control, Xining, 811602 Qinghai Province China; 2 Qinghai University Affiliated Hospital, Xining, 810000 Qinghai Province China; 3 Biomedical Research Center, Northwest Minzu University, Lanzhou, 730000 Gansu Province China

**Keywords:** Alveolar echinococcosis, *Echinococcus multilocularis*, Th1 cytokines, Th2 cytokines, Correlation analysis

## Abstract

**Background:**

Alveolar echinococcosis (AE) is a zoonotic parasitic disease caused by *Echinococcus multilocularis* larval tapeworm infections in humans that severely impairs the health of affected patients in the northern hemisphere.

**Methods:**

The expression levels of 20 cytokines associated with AE infection were measured by enzyme-linked immunosorbent assay, and the correlations between these cytokines were analysed in the R programming language.

**Results:**

Serum cytokine levels differed among individuals in both the AE patient and healthy control groups. The results of the correlations among the cytokines showed obvious differences between the two groups. In the AE patients group, Th1 and Th2 cytokines formed a more complicated network than that in the healthy control group.

**Conclusions:**

The altered correlations between Th1 and Th2 cytokines may be closely associated with AE infection, which may provide a new explanation for the essential differences between AE patients and healthy individuals.

## Background

Alveolar echinococcosis (AE) is a severe parasitic disease caused by *Echinococcus multilocularis* larval tapeworm infection in humans that is fatal if left untreated [1, 2]. The liver is the primary target of the disease and is affected in nearly 95% of cases; this disease can also spread and affect other organs, including the lungs, brain and bone [1, 3]. AE causes severe damage or dysfunction of target organs [4, 5]. This disease is restricted to the northern hemisphere, principally in rural areas of western, northern and eastern Europe; the highest disease prevalence is in central Asia, China and Kyrgyzstan [6, 7]. Epidemiological investigations have shown that pastoral regions on the Tibetan Plateau appear to be high-risk areas for AE disease due to specific landscape features and husbandry practices. Specifically, a range of different wildlife hosts, especially small mammals, are involved in the transmission of *E. multilocularis* in a pastoral region of Qinghai province [5–7].

The World Health Organization (WHO) has listed echinococcosis as one of the 17 neglected diseases targeted for control or elimination by 2050 (http://whqlibdoc.who.int/hq/2012/WHO_HTM_NTD_2012.1_eng.pdf). To date, surgery is the only potentially curative option for the treatment of AE; however, AE recurrence after hepatectomy is high, and many patients present with inoperable disease [8]. Recently, immunotherapy has been used to complement anti-infective drug approaches, and this approach was suggested to be highly effective in treating echinococcosis; however, there is no accepted immunotherapy against AE infection due to the complicated interactions between the parasites and host immunity.

The type of immune response impacts disease development, and T helper (Th) cells can selectively differentiate into the Th1 or Th2 subtype in response to an *E. multilocularis* antigen. A Th1/Th2 imbalance has been suggested to play an important role in controlling the immunological response to AE infection [9, 10]. AE patients with Th1-oriented immunity are more likely to harbour fewer parasites or even aborted parasites, whereas AE patients with Th2-oriented immunity are more likely to develop chronic AE [11]. In mice, the Th1 response was shown to dominate at the early stage of AE, and the immune response gradually shifted towards a Th2-dominated response at the late stage of AE to prevent Th1-mediated damage [11, 12]. The imbalance between Th1-type cytokines and Th2-type cytokines in AE is not completely understood due to the limited number of studies, regional differences and complex interactions between parasites and host immunological and genetic factors [9, 12].

In this study, 20 cytokines, including Th1 and Th2 cytokines, were selected according to the related literature [13–15]. The expression levels of these cytokines were compared and analysed by bioinformatics and statistical analysis methods to explore the correlations among Th1- and Th2-type cytokines in AE patients and healthy controls from Qinghai Province in China.

## Methods

### Study groups and sample collection

The participants consisted of 45 AE patients (29 females/16 males) and 45 healthy people (27 females/18 males). The mean age of the AE patients was 38 years (range, 21–52 years), and the mean age of the healthy controls was 39 years (range, 26–53 years). All the recruited participants were Tibetan and lived in the Guoluo Tibetan Autonomous Prefecture of Qinghai Province, and 91% were herdsmen. The diagnosis of AE was according to the People’s Republic of China Health Industry Standard—Diagnostic Criteria for Hydatid Disease (WS257–2006) by a professional doctor. The classification of AE patients in different clinical stages of AE was accomplished according to the World Health Organization- (WHO-) PNM (P: Hepatic localization of the metacestode; N: Extrahepatic involvement of neighbouring organs; and M: Presence or absence of distant metastases), detailed in Table 1. No patients had received any anti-inflammatory drugs or anti-parasitic drugs, and none had undergone a curative hepatectomy or a liver transplantation before the study. All healthy controls showed normal abdominopelvic cavity images as detected by B-mode ultrasound. Written consent was obtained from all participants, and this study was approved by the Ethics Committee of Qinghai Institute of Endemic Disease Control and Prevention.

**Table 1:** The classification of AE patients

Classification			Percentage (%)
Lesion numbers		Single (%)	27 (60%)
		Double(%)	14 (31.1%)
		Multiple (%)	4 (8.8%)
PNM classification	P	P1 (%)	10 (22.2%)
		P2 (%)	15 (33.3%)
		P3 (%)	18 (40%)
		P4 (%)	2 (4.4%)
	N	0	not detected
	M		undetected
Lesion classification (67 lesions in 45 patients)		Infiltrating type (%)	49 (73.1%)
		Calcification type (%)	5 (7.5%)
		Liquefied cavity type (%)	13 (19.4%)

**Note:** No adjacent organs or tissues were found to be infected in all patients; M classification was not provided due to poor medical conditions and remote areas

Five millilitres of peripheral venous blood was harvested from each participant after an 8- to 12-h fast under strict precautions in sterile tubes containing EDTA anticoagulation, and 1 mL of serum was immediately separated from the blood and preserved at − 80 °C for the measurement of cytokines.

### Serum analysis

The serum levels of 20 cytokines in AE patients and healthy controls were measured by an enzyme-linked immunosorbent assay (ELISA) kit (Thermo Scientific) according to the manufacturer’s protocols. All samples were measured three times for each cytokine, and the mean value was taken for analysis. The 20 analysed cytokines are listed in Table 2.

**Table 2:** The Th1 and Th2 cytokines analysed in the study

Cytokine type	Specific cytokines
Th1 cytokines	IL-8, IL-2, IL-12, IL-1β, IFN-γ-inducible protein 10 (IP-10), MIP-1β, MCP-1α, and IFN-γ.
Th2 cytokines	IL-4, IL-5, IL-6, IL-13, IL-18, GRO-α, and eotaxin.
Both Th1 and Th2 cytokines	Stromal cell-derived factor (SDF-1α),TNF-α, GM-CSF, MIP-1α, and regulated on activation, normal T cell expressed and secreted (RANTES).

### Literature review

We searched related studies in the PubMed database using the keywords “cytokine” and “alveolar echinococcosis” to explore data on cytokine expression in previous studies associated with AE from 1990 to 2019. After a literature review, a total of 56 studies were identified, 16 of which reported cytokine levels in human AE.

### Statistical methods

The relationships between the AE patient group and the healthy group were analysed by a principal component analysis (PCA) performed based on the Bray-Curtis distance matrix across the samples using the vegan package in R (version 3.4.4). Student’s t-test was performed to compare PC1/PC2 between groups using the stats package in R. Spearman’s correlations between cytokines were calculated using the Hmisc package in R. The correlation networks between cytokines were constructed using the GeneNet package in R and were further visualized using Cytoscape 3.4.0 [16].

## Results

### Cytokine analysis

The expression levels of 20 cytokines were compared between the AE patient group and the healthy control group, while the ELISA results showed that cytokine expression levels differed among individuals in both the AE patient and healthy control groups.

### PCA of AE patients and healthy controls

**Fig. 1:**
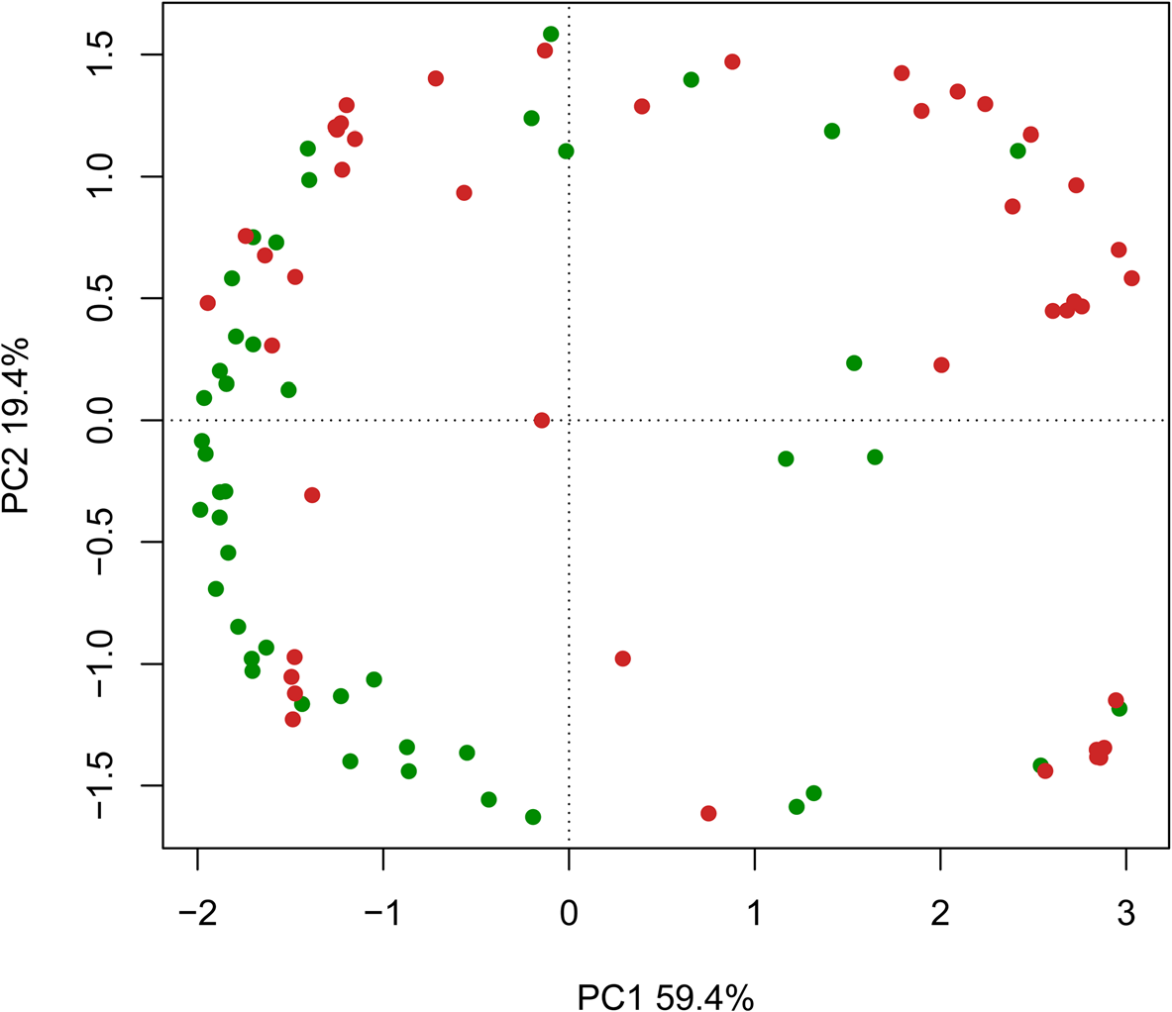
PCA of AE patients (orange) and healthy controls (green) based on the Bray-Curtis distance. The percentages of variance explained by PC1 and PC2 are 59.4 and 19.4%, respectively

**Fig. 2:**
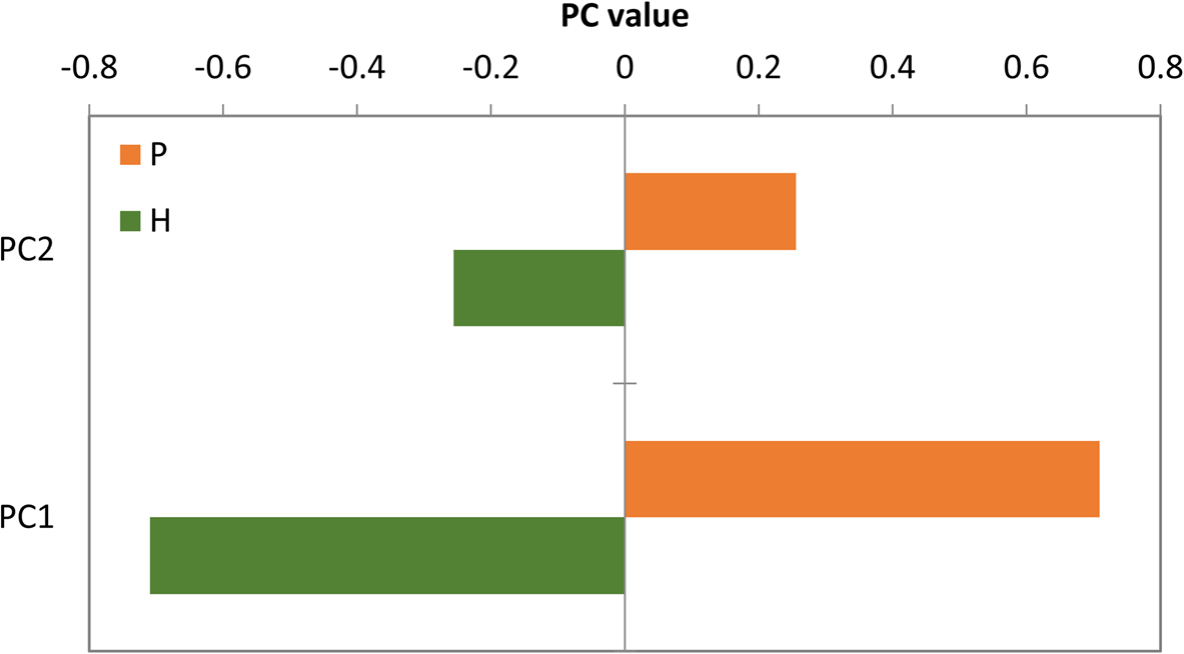
Comparison of PC1 and PC2 values between AE patients and healthy controls

The PCA showed that most samples in the AE patient group had obviously different cytokine compositions than those in the healthy control group, while a few samples showed similarity (Fig. 1). The differences in cytokine composition between the two groups were further analysed by comparing their PC1 and PC2 values (Fig. 2); these values were significantly different between the two groups (*P* < 0.001).

### Correlations between the cytokines

**Fig. 3:**
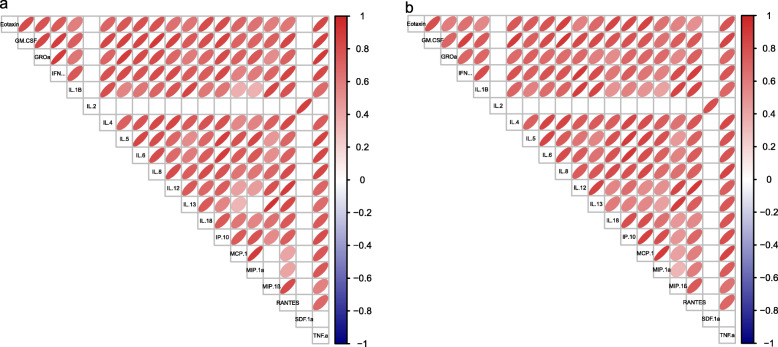
Spearman’s correlations among cytokines in the healthy controls (**a**) and the AE patients (**b**)

The correlations among some cytokines were obviously different between the two groups. In the healthy control group, there was no correlation between MIP-1α and IL-13 or among MCP-1, MCP-1α and MIP-1β (Fig. 3a). These cytokines had weak correlations in the AE patient group (Fig. 3b).

**Fig. 4:**
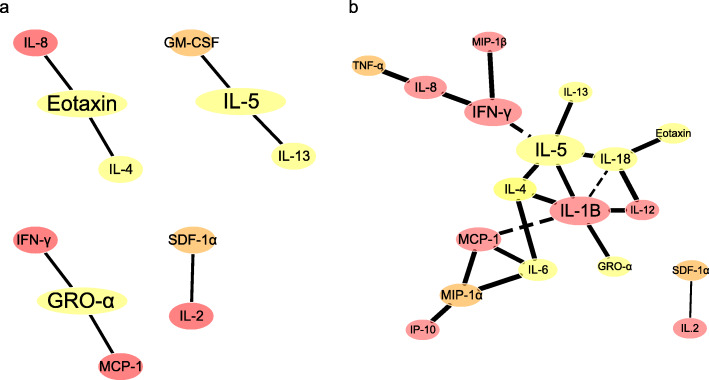
Partial correlation networks among cytokines in the healthy controls (**a**) and AE patients (**b**). Note: The colour of the node indicates the origin of the cytokine: red, Th1; yellow, Th2; and orange, both Th1 and Th2. The size of the node indicates the betweenness centrality; a node with increased betweenness centrality has increased control over the network. The solid and dotted lines indicate positive and negative relationships, respectively

A partial correlation network analysis of the cytokines confirmed the presence of more complex cytokine interactions in the AE patient group than in the healthy control group (Fig. 4a and b). In the healthy controls, the correlations were simple, and just a few of cytokines showed correlations between each other: IL-8(Th1)_Eotaxin(Th2)_IL-4(Th2); GM-CSF (Th2)_IL-5(Th2)_IL-13(Th2); (IFN)-γ (Th1)_(GRO)-α (Th2)_MCP-1α(Th1); and SDF 1α(Th2)_IL-2(Th1)(Fig. 4a). In the AE patients group, Th1 and Th2 cytokines formed a more complicated network, and more Th1 (MCP-1α, IL-1β, IFN-γ, and IL-8) and Th2 cytokines (IL-5, IL-4, IL-18) showed close correlations (Fig. 4b).

## Discussion

In the present study, the expression levels of 20 cytokines were detected to determine some differences between AE patients and healthy controls, while the results were confusing, as they differed among individuals in both the AE patient and healthy control groups. We then summarized the related literature about cytokines associated with human AE, and the results showed that the kinds of ‘different cytokines’ were not always consistent (Table 3), indicating that it is seemingly difficult to discover biomarkers for human AE at the cytokine expression level due to the complex factors in AE affecting the process.

**Table 3:** The expression levels of cytokines in *E. multilocularis*-infected humans

Cytokine type	Cytokine	Experimental type	Methods and results	Specimens	Citation
TH2	IL-10	in vitro	IL-10 levels in CD8+ lymphocytes from progressive AE patients (*N* = 12) were definitely increased after in vitro culture with crude *E. multilocularis* antigen using a protocol for intracellular staining of cytokines followed by fluorescence-activated cell sorting (FACS) analysis.	CD8+ lymphocytes cultured in vitro	Kilwinski et al. [17]
TH2	IL-10	in vitro	Peripheral blood mononuclear cells (PBMCs) isolated from progressive AE patients (*N* = 9) secreted significantly higher amounts of IL-10 than those isolated from abortive AE patients (*N* = 3); IL-10 was detected by using real-time PCR.	PBMCs cultured with Emf stimulation.	Godot et al. [18]
TH2	IL-10	clinical test	Serum IL-10 levels were significantly higher in AE patients (*N* = 40) than in healthy controls (*N* = 20), with a tendency to higher concentrations in progressive cases; IL-10 was determined by ELISA.	Serum	Wellinghausen et al. [19]
TH2	IL-5	in vitro	IL-5 production was particularly increased in PBMCs from patients with advanced AE (*n* = 14) after stimulation with crude *E. multilocularis* antigenic preparations; IL-5 was detected by RT-PCR .	PBMCs	Jenne et al. [20]
TH2	IL-5, IL-6, IL-10	clinical test	Plasma concentration levels of IL-5, IL-6, and IL-10 were slightly increased in consecutive AE patients (*N* = 28), and IL-23 concentration levels were significantly higher in AE patients; the cytokines were detected by ELISA.	Plasma	Tuxun et al. [21]
TH2	TGF-β	clinical test	Serum TGF-β levels were high, and TGF-β was expressed by most of the infiltrating lymphocytes in progressive AE patients (*N* = 18) by means oOf immunochemical staining of liver sections.	Surgical biopsy specimens	Zhang et al. [22]
TH2	TGF-β	in vitro	Higher levels of TGF-β were observed in PBMC supernatant after exposure to *Em* vesicular fluid (VF) than that from healthy blood donors; TGF-β was detected using flow cytometry and ELISA, respectively.	PBMCs supernatant	Bellanger et al. [23]
TH2	TGF-β	in vitro	A significant increase in TGF-β production was induced in PBMCs from healthy blood donors after exposure to *Em-*VF and Toll-like receptor agonists by using Multiplex Luminex® bead technology.	PBMCs exposure to *Em-*VF and Toll-like receptor agonists	Bellanger et al. [24]
TH1	IL-8, MCP-1	in vitro	Peripheral blood cells isolated from AE patients (*N* = 30) induced significant IL-8 and MCP-1 production when cultured with viable proliferating *E. multilocularis* metacestode (*Em*) vesicles; IL-8 and MCP-1 were detected by ELISA.	PBMCs cultured with Em vesicles	Dreweck et al. [25]
TH1	IFN-γ	clinical test	The mean concentration of IFN-γ in serum from AE patients (*N* = 23) was higher than that in control group; IFN-γ was detected by double antibody sandwich.	Serum	Shi et al. [26]
TH9	IL-9	in vitro	Th9-related cytokine IL-9 mRNA levels were both elevated in PBMCs and in hepatic lesion and paralesion tissues in AE patients (*N* = 14); IL-9 mRNA levels were detected by real-time PCR.	PBMCs and liver tissues	Tuxun et al. [10]
Th17	IL-17	clinical test	The plasma levels of the proinflammatory cytokine IL-17B and its soluble receptor sIL-17RB were significantly elevated in AE patients (*N* = 93); IL-17B was detected by ELISA.	Plasma	Lechner et al. [27]
Th17	IL-23	in vitro	IL-17A and IL-23 mRNAs levels were significantly elevated in the PBMCs isolated from AE patients (N = 30), and the levels were detected by real-time PCR.	PBMCs	Tuxun, et al. [28]
TH2 and TH1	IL-10, IFN-γ	in vitro	Emf-stimulated mononuclear cells from the central part of the granulomatous lesions secreted more IL-10 (TH2) and less IFN-γ (TH1) than cells from the periphery of the granuloma in progressive AE patients (N = 1); the cytokines were detected by ELISA.	Emf-stimulated mononuclear cells	Harraga et al. [29]
TH2 and TH1	IL-31, IL-33, IL-27, SDF-1, eotaxin	in vitro	The spontaneous cellular release of TH2-type cytokines IL-31 and IL-33 was clearly depressed in patients with cured, stable, and progressive AE (*N* = 57), whereas the levels of the TH1-type cytokine IL-27, anti-inflammatory cytokine SDF-1, and eotaxin increased with disease progression; the cytokines were detected by ELISA.	PBMC culture supernatants	Huang et al. [30]
Both TH1 and TH2	MIP-1α, MIP-1β, RANTES, GRO-α	in vitro	The production of CC and CXC chemokines, which associate with inflammation (MIP-1α/CCL3, MIP-1β/CCL4, RANTES/CCL5 and GRO-α/CXCL1) was constitutively higher in PBMCs when cultured with *E. multilocularis* antigen in patients with progressive, stable or cured AE (*N* = 75) than in controls; the chemokines were detected by ELISA.	PBMCs cultured with Em antigen	Kocherscheidt et al. [31]

To further understand the differences in cytokines between the two groups, we used omics methods, and the results of PCA analysis indicated that cytokine compositions were obviously different between the two groups and that the networks of cytokine correlations were more complicated in the AE patient group than that in the healthy control group. According to the correlation network results, the weak or strong correlations between Th1 and Th2 cytokines could explain the essential differences between the AE patients and the healthy controls. E. *multilocularis* metacestodes may modulate the secretion of Th1 and Th2 cytokines by Th lymphocytes in AE patients [32], and the cytokine orientation depends on the host immune response induced by *E. multilocularis* antigens [33]. Th1 cell activation induces considerable protective immunity, which involves the initiating cytokines IFN-α and IL-12 and the effector cytokines IFN-γ and tumour necrosis factor (TNF)-α, to defend against intracellular parasitic infections [34, 35]. Th2 cytokines allow parasites to proliferate at low rates by producing high levels of IL-4, IL-5 and IL-10 [13, 14]. *E. multilocularis* antigenic preparations have been reported to induce increased IL-5 production due to the activation of CD4^+^ T lymphocytes in patients with progressive AE [20]. In the present study, the correlation between the Th2-type cytokine IL-5 and the Th1-type cytokines IFN-γ and IL-1β in AE patients may be associated with the enhanced immunological response induced by parasite infection, which indicates that an inflammatory reaction is induced in AE patients. In addition, the Th2 cytokines GRO-α and eotaxin were well controlled by the Th1 cytokines MCP-1, IFN-γ and IL-8 in the healthy control group, whereas GRO-α and eotaxin levels were poorly controlled by Th1 cytokines in the AE patient group, confirming the presence of an inflammatory response in AE patients. The altered correlations among the cytokines may explain the essential differences between the AE patients and the healthy controls.

Furthermore, this parasite can secrete proteins to regulate the host immune response and survive in humans for long periods of time, and different secreted protein profiles at different stages of AE progression may explain the complex interactions between the parasite and the host [36]. A proteomic analysis of cyst vesicular fluids in AE patients contributed to the identification of potential molecular markers for diagnostic and follow-up tools, but the mechanism underlying the interplay between secreted proteins and cytokines requires further exploration.

## Conclusions

The correlations between Th1 and Th2 cytokines were simple in the healthy control group but complex in the AE patient group. Th1 cytokines, such as IFN-γ, IL-1β and MCP-1, had high betweenness centrality in AE patients, whereas Th2 cytokines, such as GRO-α, eotaxin and IL-5, had high betweenness centrality in the healthy control group. These findings may provide a new point of view to study the significant difference between AE patients and healthy individuals. However, more studies in the future will be required to clarify the “biomarkers”, such as studies including “clinically relevant populations” (such as liver non-parasitic benign cysts, liver abscesses, and mesenteric cysts) as controls, with prospective follow-up and involving large sample sizes.

## Data Availability

Data supporting the conclusions are included within the article.

## Abbreviations

AE: Alveolar echinococcosis
ELISA: Enzyme-linked immunosorbent assay
GM-CSF: Granulocyte-macrophage colony-stimulating factor
GRO-α: Growth-regulated oncogene-alpha
IP-10: Interferon-gamma (IFN-γ)-inducible protein 10
IL: Interleukin
MIP-1α: Macrophage inflammatory protein-1α
MIP-1β: Macrophage inflammatory protein-1β
MCP-1α: Monocyte chemoattractant protein
RANTES: Regulated on activation, normal T cell expressed and secreted
PCA: Principal component analysis
SDF-1α: Stromal cell-derived factor-1α
Th cells: T helper cells
TNF-α: Tumour necrosis factor α
WHO: World Health Organization
